# Implications of Long Non-Coding RNAs in Age-Altered Proteostasis

**DOI:** 10.14336/AD.2019.0814

**Published:** 2019-08-14

**Authors:** Cristina-Sorina Catana, Catalina-Angela Crișan, Dana Opre, Ioana Berindan-Neagoe

**Affiliations:** ^1^Department of Medical Biochemistry, “Iuliu Haţieganu” University of Medicine and Pharmacy Cluj-Napoca, Cluj-Napoca, Romania; ^2^Department of Neurosciences, “Iuliu Haţieganu” University of Medicine and Pharmacy Cluj-Napoca, Cluj-Napoca, Romania; ^3^Department of Psychology, Babeș-Bolyai University, Cluj-Napoca, Romania; ^4^MEDFUTURE - Research Center for Advanced Medicine, Cluj-Napoca, Romania; ^5^Research Center for Functional Genomics, Biomedicine and Translational Medicine, Institute of Doctoral Studies, “Iuliu Hatieganu” University of Medicine and Pharmacy, Cluj-Napoca, Romania; ^6^Department of Experimental Pathology, “Prof. Dr. Ion Chiricuta” Oncology Institute, Cluj-Napoca, Romania

**Keywords:** lnc RNA, cancer metabolism, age-related diseases, proteostasis, HOTAIR

## Abstract

This review aims to summarize the current knowledge on how lncRNAs are influencing aging and cancer metabolism. Recent research has shown that senescent cells re-enter cell-cycle depending on intrinsic or extrinsic factors, thus restoring tissue homeostasis in response to age-related diseases (ARDs). Furthermore, maintaining proteostasis or cellular protein homeostasis requires a correct quality control (QC) of protein synthesis, folding, conformational stability, and degradation. Long non-coding RNAs (lncRNAs), transcripts longer than 200 nucleotides, regulate gene expression through RNA-binding protein (RBP) interaction. Their association is linked to aging, an event of proteostasis collapse. The current review examines approaches that lead to recognition of senescence-associated lncRNAs, current methodologies, potential challenges that arise from studying these molecules, and their crucial implications in clinical practice.

Age-associated diseases such as cancer, cardiovascular diseases, obesity, neurodegenerative disorders, sarcopenia and several other conditions are dictated by distinct adjustments of gene expression programs that underlie aging. Recent research indicates that through examining the modifying factors of intrinsic appearance of senescent cells we could genetically program and determine their re-entry into the cellular cycle. Thereupon, in the future, senescent cells might be programmed to get involved in the treatment of cancer and aging-related diseases [1].

Aging phenotype is represented by expanded cellular senescence, reduction of stem cell population, altered proteostasis (which activates the inflammasome - a multiprotein oligomer responsible for inflammation), change in intercellular communication and loss of telomere function [2, 3].

Maintaining cellular protein homeostasis, or proteostasis, requires correct quality control (QC) of protein-related processes: synthesis, folding, conformational stability, degradation. A complicated and flexible proteostasis network (PN) parallels these processes with molecular chaperones and their QC regulators thus avoiding age-associated proteinopathies included in ARDs [4].

These mechanisms are governed by proteins that bind RNA, DNA, as well as a diversity of long non-coding RNAs (lncRNAs), long nuclear RNAs greater than 200 nucleotides, and microRNAs (miRNAs), small non-coding RNA molecules with a length of 20-25 nucleotides that are involved in controlling target gene translation and post-transcriptional modulation of gene expression. The regulatory function of lncRNAs, which are considered powerful epigenetic regulators, has been partially revealed in embryonic stem cells (ESCs) and in induced pluripotent stem cells (iPSCs) [5, 6]. The stability and longevity of RNA molecules provide a great opportunity for non-invasive diagnosis and tumoral assessment [7]. We present multiple strategies for modulating proteostasis capacity, which may aid the urgently-needed therapies for age-dependent pathologies [5, 7-9].

The accurate relationship between lncRNAs and proteostasis can be explained, both phenotypically and molecularly, by the lncRNAs - binding proteins (RBP) interactions. These interactions are essential in performing all cellular functions and in preserving homeostasis.

RBPs, RNA binding proteins, have crucial roles in a myriad of cellular processes. The first step in analyzing their possible role includes identifying their binding partner. Also, lncRNAs’ homeostasis (lncRNAstasis) paralleled the protein disruption in cellular senescence thus maintaining the correct cellular quality control (QC). Proteostasis mechanisms support the stabilization of accurately folded proteins, the heat shock protein family, and the mechanisms for lysosome and proteasome mediated protein breakdown [10, 11].

Nuclear lncRNAs modulate transcription by recruiting transcription factors to specific regions of nuclear DNA and also for ribonucleoprotein complexes with RBP. They participate in chromatin organization, gene expression, as well as structural scaffolds of nuclear domains. These complexes control gene expression at distinct key points, illustrating the critical role of lncRNA interaction with certain proteins in order to maintain cellular hemostasis [11].

The lncRNA pRNA interacts with DNA at the specific interaction point DNMT3B to control rRNA transcription [12]. In addition, PTENP1-asRNA alpha blocks transcription of PTEN coding gene by to DNMT3A (DNA methylase) at PTEN coding gene promoter [13]. PTENP1asRNA beta positively provides post transcriptional regulation of PTENP1 mRNA [11].

The nuclear enriched transcript 1 (NEAT) recruits paraspeckles RNA-binding proteins such as PSPC1, NONO/P54NR and PSF/SFPQ, directly or in a complex manner, and also suppresses gene expression by interaction with PRC1, PRC2, JARID1B, ESET and SUV39H1, chromatin binding protein/complexes [14, 15].

The lncRNA TERC, a telomerase RNA component, maintains telomere length, while the lncRNA THRIL-hnRNPL interactions modulate (TNF) α expression [16].

Cytoplasmic lncRNAs have different functions. They can act as translation regulators via base pairing with their target mRNAs or they can influence protein expression levels by increasing and decreasing mRNA stability [17, 18]. Another function of cytoplasmic lncRNAs is modulating ubiquitination process or controlling the passage of proteins or other RNAs between the cytoplasm and the nucleus [11]. Some lncRNAs indirectly regulate protein levels by influencing the available pool of miRNAs and, thereby, affecting mRNA turnover and translation. On the other hand, some lncRNAs interact directly with mRNAs, in order to enhance or suppress their translations, or with proteins, modulating their half-life time. For instance, the lncRNA GAS5 limits the “flow” of glucocorticoid receptor (GR) from the cytosol to the nucleus inhibiting GR mediated gene expression. In addition, the maternally expressed gene 3 (MEG3) which induces the p53 translation decreases the MDM2 expression while HuR can displace the lncRNA 7SL protein and also increases the p53 expression [19, 20]. Conversely, the highly expressed lncRNA 7SL could abolish p53 translation [11].

## Classification lncRNAs according to their mechanism of action

LncRNAs are heterogeneous transcripts that are not translated into proteins or encoding for small proteins [9, 21]. They can be intergenic transcripts or large intergenic non-coding RNAs (lincRNAs), enhancer RNAs (eRNAs), or sense or antisense RNAs from the same or the opposite strand of mRNA that overlaps other genes. LncRNAs produced by RNA splicing have been revealed, such as circular RNAs (circRNAs), to derive from vestigial genes without coding potential, named pseudogene-encoded lncRNAs, from mRNA promoter regions, described as promoter-associated lncRNAs, as well as from introns, long intronic ncRNAs [22-24]. Competing endogenous RNAs (abbreviated ceRNAs), which manage RNA transcripts by competing for shared miRNAs, and circRNAs are stable and accumulate in great numbers [2, 25].

Remarkably, these lncRNAs have crucial roles in gene regulation, affecting different aspects of cellular homeostasis such as proliferation, migration or genomic stability by assembling transcriptional modulators, by base-pairing with mRNAs, by enrolling chromatin modification factors, as well as by interfering with RNA-binding proteins and leading to age-associated phenotypes relevant to multiple disease pathophysiologies associated with the aging process [2, 24, 26, 27].

Experimental evaluation of lncRNAs has clarified the importance of these biomolecules, that are not only a ?transcriptional noise?, but they perform a function elsewhere in the cell after they leave the transcription site. The non-coding transcriptome could reveal unexpected molecular activities, offering a great potential to distinguish between normal and disease states [24].

**Table 1:** lncRNAs in proteostasis.

lncRNA	ARDs	Function in proteostasis	Target gene	The cellular and molecular effects of the lncRNA	Ref.
LncRNA-MALAT1	-highly expressed in cancer; Diabetic nephropathy; -atherosclerosis; - neurodegenerative processes	Protein turnover Scaffolding Autophagy	-HMGB1; β-catenin; - B-MYB; - PDGF-BB - ATG7 (miR142-3p)	-Inhibition of Tumor Cell Apoptosis; - kidney fibrosis; restored podocytes function; - Phenotypic switching of VSMCs	[34], [77], [81], [82]
LincRNA-p21	-skin cancer -colorectal cancer -prostate cancer	Protein turnover	p53; HIF-1α β-catenin *Jun B mRNA*	cell cycle arrest; apoptosis in keratinocytes; represses translation of cancer proteins	[7], [37], [54], [83], [84]
LncRNA CND1/cyclin D1	- many cancer types; - BCL2; -breast cancer	-Protein turnover	- TLS -cyclin D1	- cell cycle regulator in cancer -benefits in breast cancer therapy	[38], [39]
LncRNA-HOTAIR	-breast, gastric, and colorectal tumors; - nasopharyngeal cancer	-Protein turnover; - Scaffold function	-PRC2 - Snurportin-1; Ataxin-1	•cell proliferation, invasion, aggression, and metastasis; inhibition of apoptosis • prevents cellular senescence	[31], [29], [42], [45]
Lnc AS Uchl1	-neurodegenerative diseases; -cancer; -auditory cortex senescence	Protein turnover	-MDM2; -UPS-related proteins: p53, p14; ARF, p27KIPI, ubiquitinated proteins, monoubiquitin, BE1, PSMA7	- intensifies translation of UCHL1, which plays an important role in the UPS system	[49], [50], [51]
LncRNA GAS5	-gastric carcinoma -prostate cancer	-Protein turnover; -Membrane trafficking	YBX1; E2F1; P27Kip1	-Inhibits cellular proliferation - a growth arrest lncRNA	[52], [85]
Lnc RNA PANDA	-senescence.	Membrane trafficking	*FAS; BIK; p53*	inhibits DNA-damage-induced apoptosis	[86]
Lnc ANRASSF1	-breast, osteosarcoma, colorectal, liver, bladder, renal cell carcinoma	-Membrane trafficking; -Scaffold function	PRC2	Control of proliferation, metabolism, apoptosis and senescence; histone modifications	[36], [87]
LncRNA Gadd7	- is expressed in response to oxidative stress	Membrane trafficking	TDP-43, modulates Cdk6 levels	controlling cell-cycle progression	[88]
LncRNA 7SL	-widely upregulated in cancer tissues	-Autophagy -Protein trafficking	p53; HuR	cellular senescence	[70], [36]
Lnc RNA DICER1	-ovarian cancer; - tongue squamous cell carcinoma	Autophagy	miR-675	-A key synthesis-related factor of miRNA related to tumor cell activities; - cellular proliferative and invasive capacities	[89]
LncRNA HULC	- tumor chemoresistance; -hepatocellular carcinoma	Autophagy	-COX-2 USP22/COX-2" axis; - Sirt1	Increase triglyceride and cholesterol levels in hepatoma cell	[64]
Lnc MEG3	-colorectal cancer - Huntington’s disease	Autophagy Growth arrest	MDM2; p53	-blocks apoptosis	[20]
LincRNA *H19*	-breast cancer; -human tumor growth; - Gastric Carcinogenesis	Scaffold function	-E2F1, PRC2, HuR, KSRP	-suppression of RBmRNA via miR675; -DNA methylation; cell division cycle	[102], [103], [104]
LncRNA PRNCR1	-prostate cancer -CRC	Scaffold function	- AR	- regulation of AR-dependent gene activation events-	[74], [90]
LncRNA PCGEM1	-prostate cancer	Scaffold function	- AR	-tumor type-specific super-enhancer	[74], [90]
Lnc*TERC*	-premature neural aging in terc KO mice	Scaffold function	TRF1, TRF2	- Promotion of telomere extension -controlling the survival of NSCs - prevention of premature senescence and aging	[2], [30], [91]
Lnc* TERRA*	-neural aging	Scaffold function	TRF1, TRF2	-Suppression of telomere extension -survival of NSCs	[2], [30]
Lnc ANRIL	-upregulated in prostate cancer; - myocardial infarction - hyper-cholesterolemia	-Protein turnover; -Scaffold function	-CBX7 - let-7a/TGF-β1/Smad signaling pathway	-proliferation and migration of prostate cancer cells - antisenescence function -histone modification	[41]

Abbreviations: ANRIL- antisense non-coding RNA from the inhibitor of kinase 4 (INK4); AR- androgen receptor; ARF- ADP-ribosylation factor; Lnc AS Uchl1 - ubiquitin C-terminal hydrolases L1; ATG7- autophagy-related 7; BCL2- B-Cell CLL/lymphoma 2; BIK - ; CBX7- chromobox 7 protein; -COX-2-Cyclooxygenase-2; CRC-colorectal cancer; E2F1- transcription factor that interacts directly with RB; FAS -; Gadd7- growth-arrested DNA damage-inducible gene 7; GAS5- Growth arrest specific transcript 5; HIF-1α - Hypoxia-inducible factor 1-alpha; HMGB1- High-mobility group box protein 1; MALAT1- Metastasis Associated Lung Adenocarcinoma Transcript 1; MDM2- mouse double minute 2 protein; KSRP- KH-type splicing regulatory protein; MEG3- maternally expressed gene 3; B-MYB - Myeloblastosis Viral Oncogene; NSCs- neural stem cells; PANDA- ; PDGF-BB- platelet-derived growth factor- BB; PRC2-; PRNCR1- prostate cancer non-coding RNA 1; PCGEM1- prostate cancer gene expression marker 1; PSMA7-proteasome subunit alpha type 7; RB- retinoblastoma protein; Sirt1- silent information regulator 1 protein;; *TERC*- Telomerase RNA Component*; TERRA* - telomeric repeat containing RNA; TGF-β1- ;TLS- translocated in liposarcoma protein; TRF1, TRF2- telomere repeat factors; VSMCs- vascular smooth muscle cells; UCHL1- ubiquitin carboxyterminal hydrolase 1; USP22- ubiquitin-specific peptidase 22; YBX1- Y-box binding protein 1.

## LncRNAs in proteostasis

Aging is associated with the progressive deterioration of proteostasis, a portmanteau of two words, protein and homeostasis. It encompasses competing and integrated processes that control protein biogenesis, folding, interactions, trafficking and degradation within and outside the cell. Proteostasis dysfunction, including autophagy and the ubiquitin-proteasome pathways, leads to age-related diseases (ARDs) such as Alzheimer’s disease, cancer and other degenerative disorders, being an accepted aging factor [2, 28, 29]. In line with this, we summarize proteostasis-related lncRNAs associated with protein turnover (synthesis and degradation), trafficking and autophagy (Table 1).

### LncRNAs associated with protein turnover

Protein turnover represents the balance between protein synthesis and protein degradation. This process decreases with age in all senescent organisms. Protein turnover occurs in the brain and may contribute to protein aggregation and neurodegeneration, disturbing physiological neurogenesis and synaptic plasticity [2, 30].

Protein degradation is driven by the ubiquitin proteasome pathway. Protein synthesis depends on mRNA level. The translation rate is modulated by lncRNAs indirectly by affecting the pool of miRNAs, suppressing the mRNA turnover and translation (lincRNA-ROR and linc-MD1), or through direct interaction with proteins and mRNAs, modifying their translation [2].

Perturbations of protein-RNA interactions are involved in metabolic and autoimmune diseases, cancer, neurological and muscular disorders. Many RNA-binding proteins (RBP) such as heterochromatin protein 1, male-specific lethal-1 (MSL), the catalytic subunit of MSL histone acetyltransferase (HAT) enzyme complex (MOF), deafness dystonia peptide 1 (DDP1), Trithorax-group and Polycomb-group implicated in distinct tumor stages bind lncRNAs [31].

LncRNA-MALAT. LncRNA metastasis-associated lung adenocarcinoma transcript 1 (MALAT1), a cell cycle regulator whose depletion triggers G1 or G1/S arrest by suppressing cell proliferation and growth activating senescence phenotype [32] and a high expression molecular predictor of poor survival rates in cancer, interacts with splicing regulatory (SR) protein family members. This lncRNA triggers two cell-cycle regulators, cyclins A2 and B1, and controls the oncogenic transcription of myeloblastosis viral oncogene B (B-MYB) [33, 34].

LincRNA-p21 regulates *p21* by recruiting hnRNPK and reducing cell proliferation. It also affects somatic cell reprogramming via cell senescence or apoptosis pathway [32]. This lincRNA, interacting with cadherin-associated protein, beta (CTNNB) mRNAs, encoding βcatenin via the Wnt/βcatenin signaling pathway and decreasing oxidant stress, could have antisenescent effects in doxorubicin (Dox)treated HL1 murine cardiomyocytes, where it was shown to have a high expression [35]. LincRNA-p21 is also induced by hypoxia-inducible factor 1α (HIF-1α), being able to bind this factor, and by UVB via the p53 pathway, having an important role in UVB-induced apoptosis. Urinary levels of LincRNA-p21 lncRNA may help discriminating between prostate cancer and benign prostatic hyperplasia [7, 36, 37].

LncRNA CND1/cyclin D1, a cell cycle regulator in many cancers, is transcribed from the cyclin D1 gene promoter region. It interacts with the translocated liposarcoma (TLS) protein, a sensor for the detection of DNA damage [38]. High levels of cyclin D1 expression are associated with better outcomes of adjuvant trastuzumab therapy in HER2-positive early breast cancer [39].

LncRNA ANRIL. This lncRNA, transcribed from the inhibitor of kinase 4 (INK4) locus, is the antisense non-coding RNA in INK4 (ANRIL). It interacts with both CBX7, a component of the polycomb group protein regulator of cytokinesis (PRC1), where it activates epigenetic silencing of the CDKN2A/CDKN2B loci, and SUZ12, a component of PRC2. Its down-regulation induces translation of the cell cycle inhibitors such as P14, P15 and P16. Additionally, the lncRNA MIR31HG interacts with both PRC1 and PRC2 complexes to suppress the P16INK4A expression [Ghanam]. LncRNA ANRIL is upregulated in prostate cancer, interacting with the chromobox 7 (CBX7) protein, part of the polycomb group protein regulator of cytokinesis (PRC1) protein complex [31, 40]. This lncRNA activates the proliferation and migration of prostate cancer cells through the let-7a/TGF-β1/Smad signaling axis [41].

LncRNA HOTAIR, up-regulated during the aging process, increases ubiquitination degradation of Snurportin-1 (SNUPN) and Ataxin-1 (ATXN1) by functioning as a scaffold for DAZ interacting zinc finger protein (DZIP3) and Mex-3 RNA binding family member B (MEX3B) and their corresponding substrates [11].

**Figure 1.**
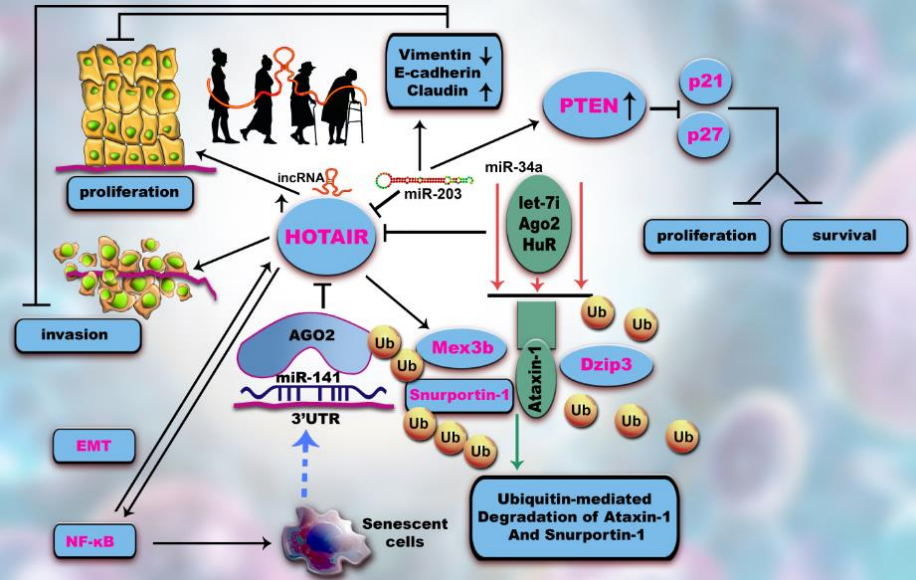
Altered HOTAIR regulation contributes to ARDs/ senescence. HOTAIR, overexpressed during aging, activates proliferation and invasion. miR-141 levels are inversely correlated with malignacy by binding to this lncRNA and thus abrogating its transcription. Both interact with/are linked to Argonaute 2 (Ago 2) complex. A positive feedback mechanism from senescent cells upregulates miR-141. The level of HOTAIR could be reduced in a micro-dependent manner by an RNA binding protein (RBP), the senescence-repressor HuR, which degrades this lncRNA. In addition, HOTAIR facilitates ubiquitination and proteolysis of Snurportin-1 and Ataxin-1. HOTAIR interacts with E3 ubiquitin ligases and with their ubiquitination substrates, Ataxin-1 and Snurportin-1. *HOTAIR* facilitates the ubiquitination of Ataxin-1 by Dzip3 and Snurportin-1 by Mex3b and accelerates their degradation. HOTAIR has a key role in cellular senescence through inducing extended expression of NF-κB target genes and also NF-κB activation during DNA damage. An NF-κB-HOTAIR axis leads to a positive-feedback loop cascade contributing to cellular senescence and chemotherapy resistance in cancers. Overexpression of miR-203 inhibits HOTAIR, triggering epithelial- mesenchymal-transition (EMT), therefore inducing cell-cycle arrest and apoptosis. The expression of phosphatase and tensin homolog (PTEN), E-cadherin and claudin is increased by blocking invasion and metastasis while p21 and p27 are downregulated.

HOTAIR is one of the first lncRNAs linked to cancer. It interacts with polycomb repressive complex 2 (PRC2), a histone methyltransferase and lysine-specific histone demethylase 1A (LSD1), an illustration of histone demethylase [31, 42, 43]. During aging, this lncRNA is degraded by the senescence repressor HuR, a miRNA-200a dependent RBP, due to its binding to the 3′UTR of *c-Jun* mRNA in a region including this miR binding site [44]. HOTAIR prevents cellular senescence through the decay of Snurportin-1 and Ataxin-1 targets via the ubiquitination pathway. Ectopic expression of lncRNA HOTAIR determines inflammation through NF-κB activation and through interleukin (IL)-6 expression [45, 46, 47]. MiRNA-203 inhibits HOTAIR, regulating tumorigenesis via the epithelial-to-mesenchymal transition (EMT) pathway [48] (Fig. 1).

Lnc AS Uchl1 (ubiquitin C-terminal hydrolase L1) intensifies translation of UCHL1, which plays an important role in the ubiquitin proteasome system (UPS) and in many other cellular processes such as differentiation, cell proliferation, as well as in brain function and in neurodegenerative diseases. Loss of function of UCHL1 leads to serious degenerative modifications in the central nervous system, this proteolytic deficit contributing to neurological conditions [49, 50]. Overexpression of UCHL1 decreased mouse double minute 2 (MDM2) levels, a factor involved in cancers, and increased the UPS-related proteins such as p53, p14, ADP-ribosylation factor (ARF), p27KIPI, ubiquitinated proteins, monoubiquitin, BE1, proteasome subunit alpha type 7 (PSMA7) and the proteasomal activity, the last five systems being implicated in auditory cortex senescence [49, 51].

LncRNA GAS5. Growth arrest specific transcript 5 (GAS5) was shown to interact with Y-box binding protein 1 (YBX1) through the GAS5/YBX1/p21 pathway, and the knockdown of lncRNA GAS5 was demonstrated to accelerate YBX1 protein turnover without affecting its gene expression. LncRNA GAS5 downregulation lowers YBX1 protein concentration, interfering with YBX1-transactivated p21 transcription and abrogating G1 phase cell cycle arrest in gastric carcinoma. The lncRNA GAS5/YBX1/p21 axis was proved to be a useful target for developing lncRNA-based treatment for cancer [52].

### LncRNAs in protein membrane trafficking

Membrane trafficking is the cornerstone of molecular biology. It compartmentalizes cells into functional recognizable units for signal initiation and processing. It is generally accepted that deregulated membrane trafficking leads to pathological aging. Kes1/Osh4, a member of the oxysterol binding protein-related protein (ORP) superfamily, and other ORPs, activate cell-cycle control functions, inhibiting phosphatidylinositol transfer protein (Sec14)-dependent membrane trafficking using the trans-Golgi (TGN)/endosomal network, inhibiting the G_1_/S transition, when cells are under caloric restriction (CR). Therefore, replicative aging is encouraged. Kes1-dependent cell-cycle control depends on the Greatwall/MASTL kinase ortholog Rim15 and is in opposition to the Sec14 action in a mechanism independent of Kes1/Sec14 total membrane-trafficking actions. ORPs define a family of stage-specific cell-cycle regulation factors with tumor suppressor-like functions [53].

Cell cycle is strictly regulated by cyclin-dependent kinases (CDKs) and several related pathways such as p53 and the retinoblastoma protein (pRB). Current research on lncRNAs outlines their involvement in the control of key cell cycle regulators such as p53, pRB, cyclins, CDKs, and CDK inhibitors. These lncRNAs are epigenetic regulators and transcription and post-transcription regulators for primary control cellular levels of cell cycle modulators through different mechanisms. Sometimes, certain lncRNAs are induced by DNA damage, leading to cell cycle arrest or apoptosis as a response to DNA damage. Consequently, deregulations of lncRNAs are involved in tumoral genesis and in chronic inflammation and they could represent possible molecular targets for both cancer diagnosis and therapy [54].

LncRNA-P21-associated ncRNA DNA damage-activated (PANDA) is specifically induced by DNA damage through the p53 pathway, through binding the nuclear transcription factor Y subunit α (NF-YA). Its activation is prevented and the expression of proapoptotic genes is suppressed. The interaction between NF-YA and p53 disrupts the cell cycle and senescence [8, 53].

Lnc RNA GAS5 is a growth arrest lncRNA involved in human malignancies. It inhibits the transcription of glucocorticoid receptor (GR) by blocking this nuclear receptor in the cytoplasm [52, 55]. Later on, it was shown to have a role on mESC proliferation. LncRNA Gas5 has a key role in controlling iPSC reprogramming, self-renewal and pluripotency of mESCs. The knockdown of Gas5 facilitates endodermal differentiation of mESCs and reduces the efficiency of iPSC reprogramming through the Dicer-miR291a-cMyc axis. It is also involved in the DNA demethylation course in mESCs [5].

ANRASSF1. This lncRNA forms an RNA/DNA hybrid at the transcriptional start site of RASSF1A, a gene encoding the Ras association domain-containing protein 1. It becomes hypermetilated during aging. Ras proteins, members of a superfamily of GTP-ases, have a key position in numerous signaling networks, counting the IIS action, controling proliferation, metabolism, apoptosis and senescence. The hyperactivation of Ras or mutant Ras proteins is difficult to target (the intrinsic enzyme activity becomes defective and it freezes them in a highly active oncogenic GTP-bound state) [36].

Gadd7. It supervises cell growth and the G1/S checkpoint induced by oxidative stress and DNA damage, destabilizing CDK6 mRNA through direct association with TAR DNA-binding protein 43 (TDP-43). This leads to cell senescence and it could be a possible biomarker for frontotemporal lobar degeneration (FTLD) [52, 56, 57].

7SL. This widely expressed lncRNA in cancer cells is involved in cell proliferation and is an integral component of "signal recognition protein" (SRP) [58]. 7SL interacts with RBP HuR, promoting translation of p53, the most important growth regulator and tumor suppressor protein [59].

### LncRNAs in autophagy

Autophagy is a versatile and protective degradation process supervising cellular quality control during the aging process [60]. The autophagic flux depends on direct improvement in somatic conservation and proteostasis. Therefore, the intracellular proteostatic signalling pathways are involved in transfering autophagic status between cells and tissues, controlling ARDs on a systemic level [61]. Certain lncRNAs were recently found to control autophagy.

H19, a suppresed lncRNA in patients with high blood sugar and diabetic cardiomiopathy, abolishes autophagy by repressing a GTPase DIRAS3, a tumor suppresing gene, therefore regulating ATG7 gene expression [61, 62].

LncRNA DICER1 - antisense RNA 1 (AS1) has an important role in autophagy and tumoral progression. Overexpressed in osteosarcoma cells, this lncRNA knockdown could suppress autophagy by inhibiting the expression levels of certain proteins as follows: autophagy-5 (ATG5), microtubule-associated protein light chain 3 (LC3-II) involved in autophagosome membrane expansion, and beclin 1, an apoptotic promoter. Moreover, miR-30b targets 3'-UTR of DICER1-AS1 and ATG5 [63].

**Figure 2.**
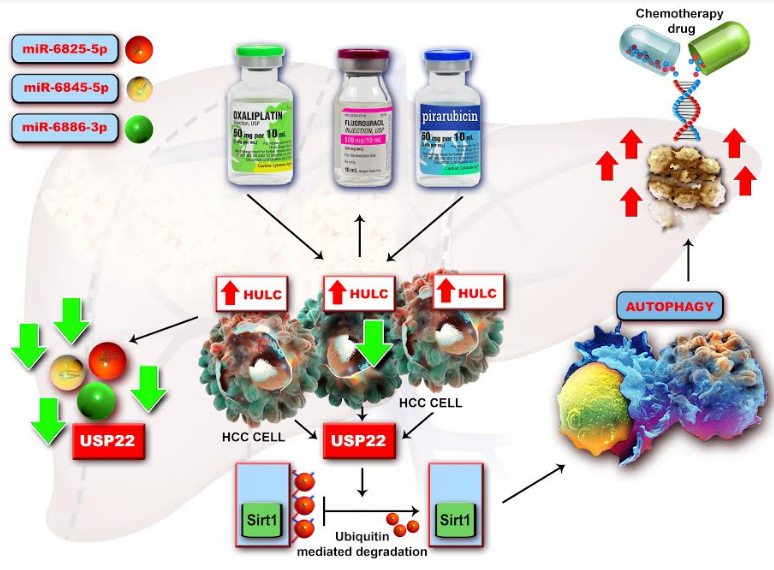
Mechanism by which lncRNA HULC activates tumorigenesis. Abbreviations: CLOCK- circadian locomotor output cycles kaput; E2F1-transcription factor involved in cell cycle regulation and apoptosis; HCC- hepatocellular carcinoma; HIF-1α- hypoxia-inducible factor 1-alpha; HMGA2- high mobility group A protein 2; HULC- highly up-regulated in liver cancer; PRKACB- protein kinase cAMP-activated catalytic subunit beta; PTTG1- pituitary tumor transforming gene; siRNA- small interfering ribonucleic acid; TWIST- the basic helix-loop-helix transcription factor ; YAP- yes-associated protein 1. lncRNA HULC, highly expressed in liver cancer, modulates the oncogene HMGA2 to activate tumorigenesis and interacts with the CLOCKmRNA, leading to the enhancement of its transcription. HMGA2 plays an essential role in the genesis of lung cancer, gastric cancer and colorectal carcinoma. HULC could be considered a molecular sponge which sequester certain miRNAs such as miR-186, miR-107 as well as miR-372, therefore reducing the translational repression of HMGA2, E2F1 and PRKACB. The expression level of HULC is positively correlated with HMGA2 and opposite to miR-186. In human HCC tissues, HULC upregulated HMGA2 expression via sequestering miR-186 promotes tumorigenesis. Moreover, HULC induces the expression of cyclin A and IL-15 in a dose-dependent manner. In HCC, HMGA2 is inhibited by miR-107 and let-7 miR-107 in breast cancer as well as siRNA as a consequence of HULC inhibition.

LncRNA HULC. Considerable research has revealed that autophagy is a key factor in tumoral chemoresistance and that lncRNA HULC is highly induced in liver cancer by therapy with antitumoral reagents such as oxaliplatin, 5-fluorouracil and pirarubicin (THP), which leads to protective autophagy. In human HCC tissues, the mechanism is mediated by the silent information regulator 1 (Sirt1) protein, the level of HULC being positively correlated with that of Sirt1. The pathway ‘HULC/ubiquitin-specific peptidase 22 (USP22)/Sirt1/protective autophagy’ increases HCC cells sensitivity to chemotherapeutic agents. This pathway could be a novel target for sensitizing HCC cells to HCC chemotherapy [64]. Mechanistically it was found that HULC could act as a molecular sponge of miR-372, 107 and 186 thus promoting tumorigenesis [65] (Fig. 2). This lncRNA increases expression of becline-1, an* autophagy* related gene, and also the interplay between LC3 and ATG3 during hepatocarcinogenesis [66].

LncRNA MEG3. The lncRNA MEG3 gene was shown to be involved in colorectal cancer, controlling certain cellular and molecular processes such as autophagy and growth arrest by suppressing MDM2, upregulating p53 and blocking apoptosis [20, 67, 68]. This lncRNA could be a novel biomarker for predicting clinical outcome in cancer [69].

LncRNA 7SL. 7SL-depleted cells are lead to cellular senescence and autophagy due to the competitive binding between HuR and 7SL, which can be removed, increasing the p53 gene expression. It also blocks the cell cycle and enhances senescence and autophagy [70].

Other examples of lncRNAs involved in controlling all autophagic stages are HOTAIR, MALAT1, NBR2, PTENP1, and recently NEAT1 activating autophagy in Parkinson’s disease via PINK1 protein [71, 61]. Equivalently, lncRNAs GAS5 and CAIF modulate ATG3 in certain pathological conditions such as osteosarcoma, myocardial infarction and cancer [72, 61].

### LncRNAs: scaffold function

LncRNAs could serve as protein scaffolds, participating in the assembly of ribonucleoproteins that link the factors together to produce new functions. The association between lncRNAs and disease may involve their scaffolding capacity. Certain lncRNAs present specific protein-binding domains that incorporate each molecule together. This action may have an impact on transcription or repression processes [73, 74].

LincRNA *H19* controls a collection of genes consisting of H19 and insulin-like growth factor-2 (IGF2) through the interaction with methyl-CpG-binding domain protein 1 (MBD1). Therefore, a ribonucleoprotein complex H19-MBD1 is formed. It represses gene expression by recruitment of histone lysine methyltransferases. Both H19 and IGF2 are involved in aging. Moreover, their increased level promotes ARDs [75].

LncRNAs PRNCR1 and PCGEM1. Two lncRNAs, namely prostate cancer non-coding RNA 1 (PRNCR1) and prostate cancer gene expression marker 1 (PCGEM1), generally overexpressed in the most aggressive forms of prostate cancers, precisely bind to the androgen receptor (AR) and strongly amplify androgen receptor-mediated gene expression in both ligand-independent and dependent pathways [75].

**Table 2:** Senescence- associated lncRNAs and neurodegenerative disorders.

lncRNA/expression	Implication in neurodegenerative disorders	Abnormalities in neuronal process/ Clinical features	Reference
MEG3 -expressed in the nucleus and cytoplasm	-upregulated in the hippocampus of old mice; -downregulated in old induced striatal medium-sized spiny neurons (MSSNs); - PTEN/PI3K/AKT signaling cascade	-cognitive decline -downregulated in HD brain tissue - synaptic plasticity in neurons	[30], [92]
SORL1-AS	- upregulated in AD disease brain affecting Aβ formation	-AD; -Protein aggregation; -cognitive impairment	[30], [93]
Six3OS -spatiotemporal expression	- Regulation of Six3 targets through interactions with Eya proteins and the chromatin-modifying protein Ezh2;	- adult mouse neurogenesis	[94]
17A	-upregulated in frontal and temporal cortices -increases Aβ secretion	-AD; -Abolish GABA B2 intracellular signaling	[95]
MALAT-1	- upregulated in human aged SVZ; -upregulated in the hippocampus of old mice; - scaffold for proteins and RNAs	-cognitive decline; -neurodegeneration; -PD	[30], [96], [97]
UCHL1-AS	-downregulated in murine dopaminergic cells; - regulated by a transcription factor Nurr1 required for dopamine cells differentiation	- Neurodegeneration; -PD	[50]
ANRIL	-altered expression in all tissues	-AD; - Neurodegeneration;	[98]
HOTAIR	- high expression of HOTAIR promotes PD	-PD	[99]
BACE1-AS	- Increases BACE1 mRNA stability and Aβ42 formation	- up-regulated in AD brains	[100], [101]

Abbreviations: Aβ - amyloid β; AD- Alzheimer’s disease; ANRIL- antisense non-coding RNA from the inhibitor of kinase 4 (INK4); GABA - gamma-aminobutyric acid; HD- Huntington’s disease; HOTAIR - Hox transcript antisense intergenic RNA; MALAT-1- Metastasis Associated Lung Adenocarcinoma Transcript 1; MEG3- maternally expressed gene 3; MSSNs- medium-sized spiny neurons; NURR1- Nuclear receptor related 1 protein; PD- Parkinson disease; PI3K - phosphatidylinoside-3-kinase; PTEN- phosphatase and tensin homolog; Six3OS - Six3 opposite strand ; SORL1-AS- sortilin related receptor antisense transcript; SVZ -subventricular zone; UCHL1- ubiquitin carboxyterminal hydrolase 1; Vax2OS- ventral anterior homeobox 2 opposite strand.

After the interaction between PRNCR1 and AR, the association of disruptor of telomeric silencing 1 like histone H3 methyltransferase (DOT1L) to the PRNCR1-AR complex is accelerated through acetylation at the C-terminal of AR protein. DOT1L mediates N-terminal acetylation of AR protein, which increases the enrollment of lncRNA PCGEM1. In prostate cancer cells, translation of short hairpin RNA targeting these two lncRNAs was shown to actively suppress proliferation of cancer cells and tumor growth in murine models [76].

LncRNA MALAT1. The downregulation of MALAT1 decreased platelet-derived growth factor-BB (PDGF-BB)-induced proliferation and migration by inhibiting autophagy. MALAT1 functions as a competing endogenous RNA (ceRNA) controlling autophagy-related 7 (ATG7) gene transcription via sponging miR142-3p. It switches the phenotype of vascular smooth muscle cells (VSMCs) with consecutive proliferation, contributing to different vascular conditions such as atherosclerosis, transplant vasculopathy, in-stent restenosis, or vein bypass graft failure [77].

## LincRNA HOTAIR

LncRNAs - Telomerase RNA Component* (TERC*) and telomeric repeat containing RNA (*TERRA)* are telomerase limiting factors maintaining telomere length and controlling the survival of neural stem cells (NSCs) in neural aging [2,30] (Table 2). LncRNA *TERC* provides a template for the biosynthesis of telomeric units and forms a complex with other proteins. In addition, this lnc has a catalytic function through adding telomere repeats [78]. Dysregulation of TERRA leads to premature aging; elevated levels in particular result in a specific syndrome consisting of immunodeficiency, facial dysmorphism and centromeric instability [79, 80] (Fig. 2).

## Conclusions

Aging is governed by important adjustments in protein expression patterns modulated by lncRNAs, which critically modify both the pathological and physiological decline associated with senescence. Their potential usefulness in cancer or neurodegenerative diseases is not fully clarified at present. However, we can see the refined mechanisms involving the regulatory interaction between lncRNAs, miRNAs and RBPs as key actors which could represent novel targets for future therapeutic interventions.

In summary, this analysis on lncRNAs has revealed, through a deeper molecular undestanding, that they are truly age-related functional biomolecules with a vital contribution in normal physiology or aging-associated dysfunction.
